# Supplementary material for: Deep learning-based near-field effect correction method for Controlled Source Electromagnetic Method and application
Source: PLoS One. 2024 Nov 11;19(11):e0308875. doi: 10.1371/journal.pone.0308875 (PMC11554044; doi:10.1371/journal.pone.0308875)
Supplement: Attachment — Submitted filename: Response to Reviewers.docx [file pone.0308875.s002.docx]

*Dear Dr. Donta:*

*Thank you for the academic editor and reviewer's comments concerning our manu entitled "Deep learning-based near-field effect correction method for Controlled Source Electromagnetic Method and application". Those comments are all valuable and very helpful for revising and improving our paper, as well as the important guiding significance to our researches. We have studied comments carefully and have made correction which we hope meet with approval. Revised portion are marked in blue and yellow in the paper. The main corrections in the paper and the responds to the reviewer's comments are as flowing:*

***1.*** *The literature of this paper is weak. It is recommended to consider most recent studies and discuss their limitations. It is also requested to provide which of these limitations are addressed in this paper.*

***Response****:Thank you for your comments. We supplemented the introduction section with a description of the existing controllable-source EM method of near-field correction and discussed its limitations, such as increasing the cost of the work and decreasing the effective detection depth. The method in this paper, however, can effectively avoid the above problems and does not require additional emission sources or deletion of low-frequency data.*

2. *The discussions related to the plots, such as possible reasons noticed during experiments to gain superior performance of the proposed work over others are listed. Primary benefits, challenges, limitations are discussed.*

***Response：****Thank you for your comments. You are right that we have discussed the plots as well as the experiments extensively in the paper, and have made further modifications and additions to the original. For example, in subsection 3.1, we have added a discussion on the fact that the more complex the model is, the more difficult it is to calibrate it. At the same time, we have fully revised 5.Conclusion and Discussion sections to make the language more condensed and streamlined.*

3. *Some of the mathematical formulae are available in published papers. For example, Eq. (7 and 8) available in published works maybe in Land subsidence prediction using recurrent neural networks. It is recommended to acknowledge the formulae used from the published works appropriately.*

***Response：****Thanks for your comments, we checked and cited the relevant papers based on the information you provided. Also carefully checked the other formulas to make sure the same problem does not occur .*

4. *Provide the source code link (footnote or as a weblink citation) within the paper through GITHUB for a transparent and visibility for the implementations.*

***Response：****Thanks to your comments, we have uploaded the source code of our build network as well as the empirical data and other related files to GITHUB and inserted a link to it in the conclusion section of the paper (https://github.com/cxjcxj123/CSEM-LSTM_CNN). In addition, the data covered in the paper is also made public in Google Cloud Drive(https://drive.google.com/drive/folders/1d8Yfd2jvDWV0nnzfgduw8ezxflEEwdRf?usp=drive_link).*
