# Supplementary material for: Deep learning-based near-field effect correction method for Controlled Source Electromagnetic Method and application
Source: PLoS One. 2024 Nov 11;19(11):e0308875. doi: 10.1371/journal.pone.0308875 (PMC11554044; doi:10.1371/journal.pone.0308875)
Supplement: Attachment — Submitted filename: Response to Reviewers.docx [file pone.0308875.s003.docx]

*Dear Dr. Wu:*

*Thank you for the academic editor and reviewer's comments concerning our manu entitled "Deep learning-based near-field effect correction method for Controlled Source Electromagnetic Method and application". Those comments are all valuable and very helpful for revising and improving our paper, as well as the important guiding significance to our researches. We have studied comments carefully and have made correction which we hope meet with approval. Revised portion are marked in green in the paper. The main corrections in the paper and the responds to the reviewer's comments are as flowing:*

***Reviewer 4：***

1. *What kind of inversion did you do for the original CSAMT data? MT or Controlled source inversion including the transition zone effect? It the MT only, it is not a verification. If you use Dipole1D, please do full controlled source inversion to verify your results after NN transformation.*

***Response:****Thank you for your comments. In the comparison of the measured data, we performed the inversion of the CSAMT raw data after obtaining the full domain apparent resistivity, the reason for this is that the geological structure of the work area where this measured data is located is unusually complex, and the data has strong three-dimensional characteristics, so the direct inversion with the source is very ineffective. We used Dipole1D in the CSAMT forward, but the inversion did not use Dipole1D. After a long time of research, we did not find the NN transformation, sorry for that.*

2. *Why do you do the correction of the near (transition zone) effect and not the true CSAMT inversion (even 1D).*

***Response:****Thank you for your comments, there are various methods to reduce the impact of CSAMT field source effects, obviously direct CSAMT band source inversion is an effective solution, and also includes correction methods based on the definition of region-wide apparent resistivity, etc., and the correction method discussed in this paper is one of them. In addition, after correcting for near-source (overbanding) effects, the csamt data can then be processed using the inversion method of mt, which is simpler and more mature in comparison.*

3. *Why do you analyze the Kf-Kn results if all your early tests shown that this net give bad results?*

***Response:****Thank you for your comments. In the previous review comments, some reviewers mentioned that they would like us to make a comprehensive comparison between the method of this paper and the traditional method, so we not only tested the results of Kf-Kn in the theoretical model, but also made comparative analyses in the measured data, in order to obtain a more comprehensive comparison.*

***Journal Requirements:***

*Please review your reference list to ensure that it is complete and correct. If you have cited papers that have been retracted, please include the rationale for doing so in the manuscript text, or remove these references and replace them with relevant current references. Any changes to the reference list should be mentioned in the rebuttal letter that accompanies your revised manuscript. If you need to cite a retracted article, indicate the article’s retracted status in the References list and also include a citation and full reference for the retraction notice.*

***Response:****Thanks for the heads up.The problem with the reference list has been noted, and the formatting has been modified using the ‘Vancouver’ style as required by the journal, with the changes highlighted in green to ensure that the reference list is complete and correct. We have also checked that we have not used withdrawn papers.*
